# Mental Health in Somaliland: a critical situation

**DOI:** 10.1192/bji.2019.14

**Published:** 2020-02

**Authors:** Fatumo Abdi Abdillahi, Edna Adan Ismail, Swaran P. Singh

**Affiliations:** 1 Speciality Registrar in Public Health, Faculty of Public Health, London, UK. Email: Fatumoabdi7@gmail.com; 2 Founder and Dean of Edna Adan Teaching Hospital and Edna Adan University, Hargeisa, Somaliland, East Africa; 3 Head of Mental Health & Wellbeing, Division of Health Sciences, Warwick Medical School, University of Warwick, Coventry, UK

**Keywords:** Low-and-middle-income countries, stigma and discrimination, transcultural psychiatry, epidemiology, human rights

## Abstract

Somaliland is experiencing an explosion of mental health problems that has received little coverage. The country has experienced devastating civil wars that have resulted in widespread trauma, and the lack of necessary mental health infrastructure is an obstacle to allowing the population to heal and recover. War trauma, poverty, unemployment and widespread substance misuse (khat) have all negatively affected the mental health of its citizens. This report provides an overview of a rapid needs assessment carried out across Somaliland that examined current service provision, gaps in services, and interviews with mental health professionals and caregivers.

A mental health crisis of monumental proportions has engulfed Somaliland in the Horn of Africa, but it has attracted little attention globally. Somaliland lies to the north-west of Somalia on the southern coast of the Gulf of Aden and, following a civil war, declared independence from Somalia in 1991 (Bradbury *et al*, 2003). It has a population of approximately four million and is bordered by Somalia, Djibouti and Ethiopia. A study carried out across four regions estimated that at least one person in every two households had some form of mental illness (General Assistance and Volunteers Organization (GAVO), 2004), and yet just four qualified psychiatric doctors serve the population in public hospitals.

The Somaliland healthcare system has never developed beyond providing the most basic functions, which leave it ill-equipped to deal with any significant challenges (Memiah, 2015). The overall health of the Somaliland population is among the poorest in the region (United Nations Children's Fund, 2012) with a life expectancy of just 50 years. The Somaliland Ministry of Health recently launched its second National Development Plan (2017–2021), which set out its ambitious plans to develop the country (Somaliland Ministry of Health, 2017), yet mental health was noticeably absent from its main priorities. Given the scale of the problem, the government and its partners should prioritise and emphasise the need to invest in both the prevention and the treatment of mental illness across the country. The United Nations Sustainable Development Goals (2015–2030) specifically include mental health and substance use disorders (World Health Organization, 2016), and the Mental Health Action Plan (2013–2020) also sets a range of targets aimed at achieving equity through universal health coverage.

Given Somaliland's admirable efforts to rebuild and seek international recognition as a peaceful and progressive country in the region, we invite the global health community to engage with and support this work. This paper summarises findings from a mental health needs assessment undertaken in late 2017 across Somaliland.

## Determinants of mental health disorders

Hundreds of thousands of people died over a decade as two traumatic civil wars were fought from 1987 to 1996 within the country. The first civil war was initiated by the Somali government dictatorship at the time and involved tactics such as aerial bombardment, land mines, imprisonment, torture and rape. Seasonal rains still continue to unearth mass graves across the country. This intense period of conflict led to widespread displacement; much of the population experienced and witnessed high levels of violence. Continuous impoverishment of the already scarce resources, harsh droughts, widespread use of the amphetamine-based leaf khat and unemployment have further weakened the country and contributed to the increase in poor mental health. Despite these obstacles, Somaliland is considered an African success story, having independently initiated and sustained peace, and is conducting free and fair democratic elections and providing sanctuary for people fleeing areas of conflict in the region, such as Yemen, Syria, Ethiopia and Somalia. In neighbouring Somalia, the situation is equally dire, as an estimated one in three individuals are affected by mental illness (World Health Organization, 2010).

## Stigma and cultural perceptions of mental health

The Somali perception of mental health is binary; one is mad (*waali*) or not mad. The concept of a spectrum of mental illness and health simply does not exist. Once an individual is labelled as having a mental illness, the illness – and indeed the associated stigma – is considered permanent and irreversible. As a result, stigma is deeply rooted and all pervasive. Patients and their families face negative attitudes and physical harm from society, leaving many socially isolated and vulnerable.

## History of psychiatric services

**Fig. 1:**
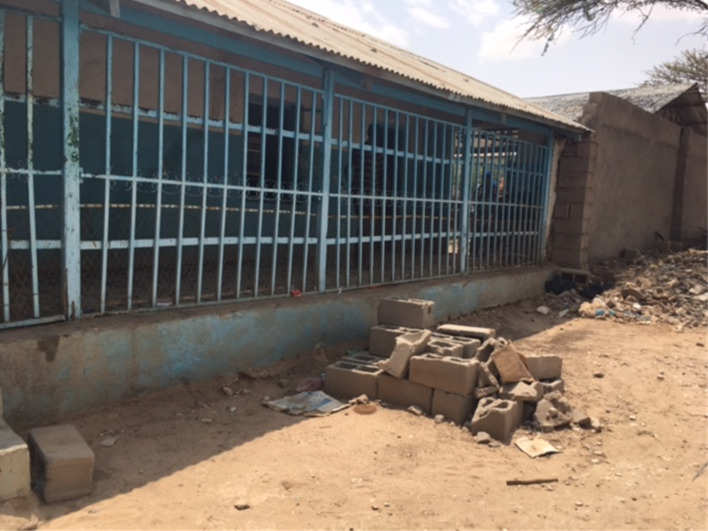
Entrance to Mental Health Department, Hargeisa Group Hospital, Hargeisa, Somaliland.

The first mental health department was established in 1948 in Berbera Hospital, which was a formerly a prison, and it was almost 25 years later that a second department was added to Hargeisa Hospital in 1971 (Fig. 1). Since 2009, three more departments were established in the towns of Borame, Gabiley and Burao.

## Organisation of current mental health services: public hospital services

**Fig. 2:**
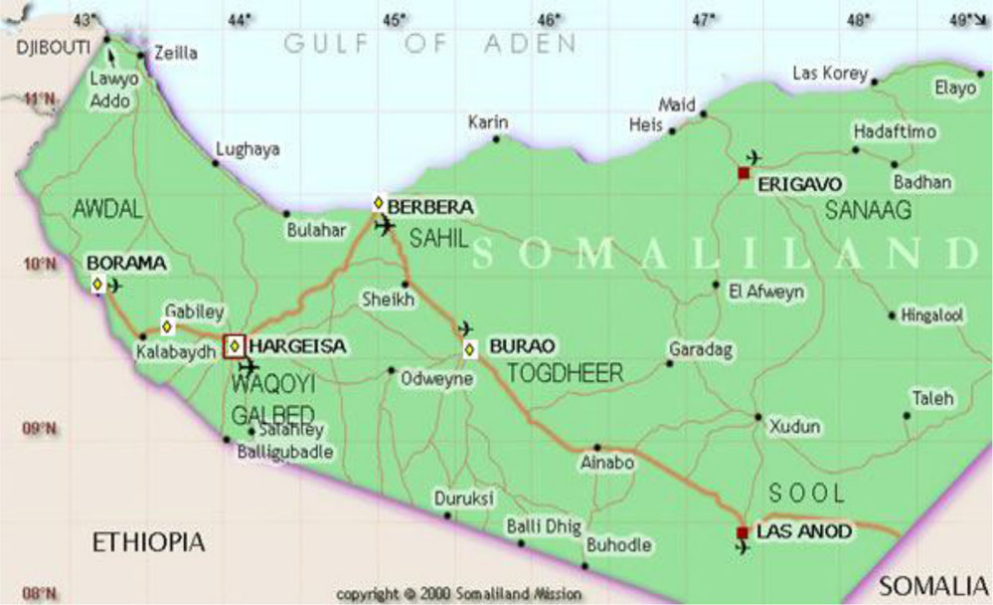
Map of Somaliland. Yellow diamonds denote the towns/cities mental health departments are located in across the country.

The Somaliland Ministry of Health is responsible for five mental health departments (all within general hospitals) located in principal towns, namely Hargeisa, Berbera, Borame, Gabiley and Burao (Fig. 2). In-patient capacity ranges from 100 beds at Hargeisa Group Hospital to just 13 beds in Burao Mandhey Hospital. All public psychiatric care is free of charge, and all departments offer limited in-patient (approximately 250 beds nationally) and out-patient services. The main mental health diagnoses reported were schizophrenia, bipolar disorder, post-traumatic stress disorder, depression, mania and generalised psychosis (acute and chronic). Substance misuse was often an additional comorbidity in men (khat and/or hashish). High relapse rates (between 30 and 50%) were attributed to the lack of available medication and poor treatment compliance due to social stigma. Accessing and maintaining contact with services is far more difficult for rural or remote communities.

Somaliland has five medical schools, and mental health training is included in the core undergraduate medical curriculum but is not generally considered a desirable speciality. Nurses and social workers are present but few in number. Lack of funds has meant that only three hospitals employ a qualified psychiatrist full time (Hargeisa, Gabiley and Borame). Staff reported lower pay than professional peers, fewer job opportunities, no career progression and poor working conditions, all of which contribute to the professional stigma reported.

## Private mental health facilities: ‘*Ilaaj’* centres

By far the most common route into mental health services involves the network of informal, unregulated, private mental health in-patient facilities available throughout the country. These are known as ‘*Ilaaj*’, which is an Arabic word meaning ‘cure or treatment’. Any citizen can open an ‘Ilaaj’. Some centres are run by traditional or faith healers, but the vast majority do not provide any level of clinical expertise to patients. Just seven of the private ‘Ilaajs’ are government-authorised facilities that provide clinic care from a psychiatrist. Patients are forcibly incarcerated by their families for a monthly fee, and are only released at their discretion. Conditions in ‘Ilaajs’ are known to be appalling, with treatment involving being forcibly chained and held in overcrowded conditions, and verbal and physical abuse (Human Rights Watch, 2015). There has been a rapid proliferation of these centres across the country, and most patients will spend time (often years) in an ‘Ilaaj’ centre, particularly in the early stages of illness. No data is available on the number of ‘Ilaajs’ in operation, and they are not subject to any legal frameworks or governance mechanisms.

## Mental health legislation and policy

There are no mental health legislations in place to protect the lives, rights and integrity of people with mental illness. A comprehensive Somaliland National Mental Health Policy was published in 2012 (Somaliland Ministry of Health, 2012) and clearly sets out how services could be developed and organised, including the development of community-based services, training, research and legislation. A lack of funds and political will has meant that it has yet to be implemented.

## Service gaps

There are no community-based mental health services, leaving families to cope with their ill relatives and the associated social stigma alone. There are no drug and alcohol rehabilitation or child and adolescent services available (including for those with special needs). With little or no knowledge of mental health disorders and appropriate treatment, families often use metal chains to confine family members (including children). Challenges also include the absence of health infrastructure, reliable funding, legal protection and equity and community-based mental health, poor access to medication and insufficient numbers of trained specialists.

## Moving forward

Somaliland's lack of recognition as an independent country means that donors give to civil society; consequently, priorities may be set but the government does not control the money from donors. Current services are therefore sparse and inadequate to deal with the immense need. An international multidisciplinary approach is essential to addressing a problem on this scale. Support is needed in areas such as the development and implementation of a long-term national strategic plan for mental health alongside policies, guidelines and legislation. Ensuring sustainable, long-term financial support, post-graduate specialist training, regulation of services, and improved supply of and access to psychotropic drugs are all essential.

There are more than 336 functional health facilities across the country, of which 296 are primary health units or mother and child health/health centres, which might serve as hubs for community-based mental health services. Primary health units are staffed by at least one trained community health worker with an emphasis on prevention of disease and health promotion. Health centres are normally staffed by qualified nursing staff.

As this is a fragile, post-conflict, self-declared state, the Somaliland government has placed great emphasis on attracting investment and building economic stability, but it has yet to focus on the health of its population and recognise the explosion of mental health disorders and how this could affect its future. This paper invites the support of the global health community to assist the country to develop mental health services through appropriate policy formulation, community enlightenment, infrastructure improvement and capacity building of frontline providers to deliver mental healthcare that is patient centred, evidence based and culturally aligned.
